# Fake news in neglected tropical diseases: The case of neurocysticercosis

**DOI:** 10.1371/journal.pntd.0008208

**Published:** 2020-06-18

**Authors:** Héctor H. García, Oscar H. Del Brutto

**Affiliations:** 1 Cysticercosis Unit, Instituto Nacional de Ciencias Neurológicas, Lima, Perú; 2 School of Medicine, Universidad Espíritu Santo–Ecuador, Samborondón, Ecuador; Istituto Superiore Di Sanita, ITALY

## Viewpoint

According to the Cambridge Dictionary (Cambridge University Press), fake news are false histories that appear to be news and are spread on the internet or other media, usually created to influence political views or as a joke. Media coverage of medical matters also occasionally adds fake news generated by ignorance or sensationalism. The recent death of the famous Mexican television star, Sebastián Ferrat, due to neurocysticercosis led to misinformation disseminated by the media regarding how this disease is acquired.

**Fig 1:**
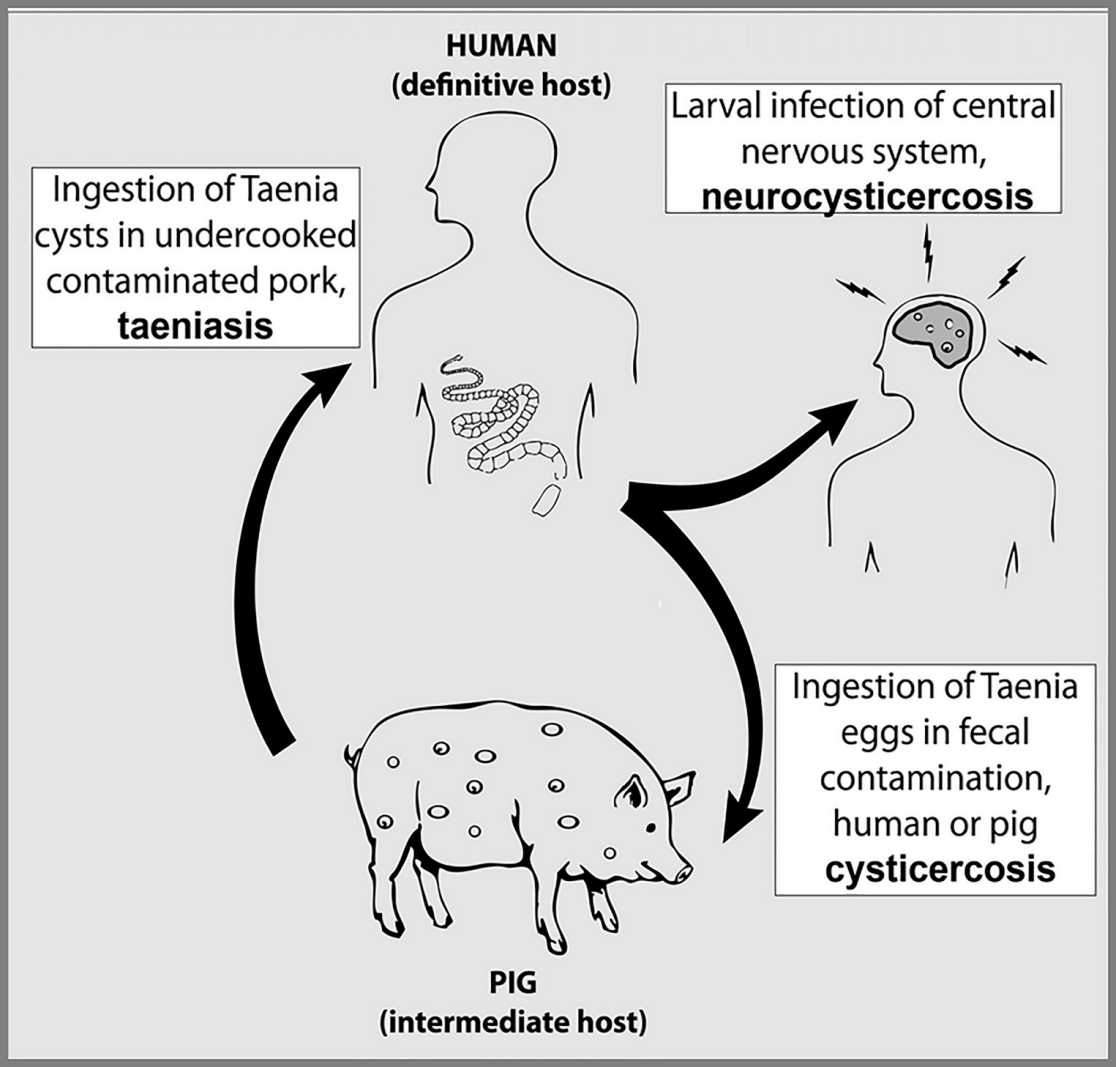
Life cycle of *Taenia solium*. Humans are the only hosts for the adult tapeworm and the sole source for cysticercosis infection in humans and pigs. *Reproduced from O'Neal SE et al*, *Ring-screening to control endemic transmission of Taenia solium*. *PLoS Negl Trop Dis*. *2014 Sep 11;8(9)*:*e3125*. *doi*: *10*.*1371/journal*.*pntd*.*0003125*. *eCollection 2014 Sep*.

Neurocysticercosis, the most common helminthic infection of the central nervous system, kills thousands of individuals per year worldwide. While pigs are part of the life cycle of the tapeworm *Taenia solium*, the causal agent of cysticercosis, human and porcine cysticercosis is only acquired by ingestion of microscopic tapeworm eggs through fecal–oral contamination, not by eating contaminated pork [1,2]. Humans are the only definitive host harboring the adult tapeworm stage (acquired by ingesting poorly cooked pork with cysts) and the sole source of cysticercosis infections in humans and pigs (Fig 1). On its coverage of the recent case, media suggested that 2020 is the year to give up bacon and even coined a new English word: “Aporkalypse”[3]. Other prestigious broadcasting media, such as the Chicago Tribune [4], also blamed ingestion of “bad pork” as the cause of cysticercosis.

Obviously, journalists mixed up the role of pigs in human cysticercosis. This misinformation confuses millions of lay people who turn to avoid pork consumption instead of being aware of the actual cause of disease acquisition (close contact with asymptomatic *Taenia* carriers who transmit the disease).

The same misconception had also reached the awarded television series House M.D. years ago. In the pilot of this series—titled “Everybody lies”—seven million people watched how the main protagonist diagnosed neurocysticercosis in a young Jewish woman who had a seizure attack, on the basis of her having ham in her fridge despite being Jewish. According to the script, this was the most important clue that led to the diagnosis (in other words, that women acquired the disease by eating ham). The title of the episode also referred to the fact that the patient denied an important diagnostic clue and lied about it because of religious reasons.

As a public health measure, media producers should be encouraged to get expert advice on technical issues to avoid misinformation that can have a negative impact on the general population.
